# From Noordwijkerhout to Bendigo: lessons learnt in developing a high risk foot clinic in regional Australia

**DOI:** 10.1186/1757-1146-6-S1-O31

**Published:** 2013-05-31

**Authors:** Byron Perrin, Marcus Gardner

**Affiliations:** 1 La Trobe Rural Health School, La Trobe University, Bendigo, Victoria, 3550, Australia; 2 Lower Extremity Gait Studies Program, La Trobe University, Bundoora, Victoria, 3086, Australia; 3 Bendigo Health, Bendigo, Victoria, 3550, Australia

## Background

In 2003 a podiatrist from Bendigo Health attended the 4th International Symposium of the Diabetic Foot in Noorwijkerhout, the Netherlands. Since then the regionally-based, outpatient, multi-disciplinary Diabetic Foot Clinic (DFC) was developed.

## Methods

On establishment, the overarching goal of the DFC was to provide the best evidence-based care possible to those at the highest risk of developing diabetes-related foot problems in the region. To achieve this, the DFC undertook a ten-year process of continual quality improvement activities that included a series of retrospective clinical audits. This paper describes the results of these audits.

## Results

The DFC has a staffing profile similar to that of an intermediate model high-risk foot clinic (IDF 2005). The proportion of patients classified as high risk has significantly increased from 43% in 2003 to 91% in 2012. The number of wounds managed in the DFC has more than doubled, and wound healing rates have improved from an average of 110 (SD 102) days in 2003 to 71 (SD 73) days in 2012. The DFC has also developed an important leadership role in promoting the use of evidence-based practice in the region.

## Conclusion

The DFC is a modest high-risk foot clinic that has vigilantly collected clinical data to inform clinical practice and service planning. Despite weaknesses in acute care, the DFC is achieving excellent outpatient clinical outcomes. This reporting of ten years of experience by the DFC may provide valuable information to other health services that aim to improve the foot-health of people with diabetes.

